# Integrating data on the *Arabidopsis* NPR1/NPR3/NPR4 salicylic acid receptors; a differentiating argument

**DOI:** 10.3389/fpls.2015.00235

**Published:** 2015-04-10

**Authors:** Xiahezi Kuai, Brandon J. MacLeod, Charles Després

**Affiliations:** Department of Biological Sciences, Brock University, St. Catharines, ONCanada

**Keywords:** NPR1, *Arabidopsis*, salicylic acid, receptor, plant immunity

## Abstract

Salicylic acid (SA) is a mandatory plant metabolite in the deployment of systemic acquired resistance (SAR), a broad-spectrum systemic immune response induced by local inoculation with avirulent pathogens. The NPR1 transcription co-activator is the central node positively regulating SAR. SA was the last of the major hormones to be without a known receptor. Recently, NPR1 was shown to be the direct link between SA and gene activation. This discovery seems to be controversial. NPR1 being an SA-receptor is reminiscent of the mammalian steroid receptors, which are transcription factors whose binding to DNA is dependent on the interaction with a ligand. Unlike steroid receptors, NPR1 does not bind directly to DNA, but is recruited to promoters by the TGA family of transcription factors to form an enhanceosome. In *Arabidopsis*, NPR1 is part of a multigene family in which two other members, NPR3 and NPR4, have also been shown to interact with SA. NPR3/NPR4 are negative regulators of immunity and act as substrate adaptors for the recruitment of NPR1 to an E3-ubiquitin ligase, leading to its subsequent degradation by the proteasome. In this perspective, we will stress-test in a friendly way the current NPR1/NPR3/NPR4 model.

## New Insights into SA Signaling

Salicylic acid (SA) is an endogenous plant hormone essential to the deployment of a long-lasting, broad-based immunity termed systemic acquired resistance (SAR). SA protects plants from a wide range of phytopathogens by mediating immune response at both local and systemic level (Vlot et al., 2009). SA has also been found to participate in abiotic stress responses. For instance, exogenous SA applications induce tolerance to copper toxicity (Mostofa and Fujita, 2013). In addition to its role in biotic and abiotic stress resistances, SA can influence plant flowering and thermogenesis (Vlot et al., 2009). Due to its biological significance, the synthesis and signal transduction of SA has been intensely studied. Still, not much is known about the molecular details of the SA signaling pathway and the SA receptor remained unidentified for decades.

In 2012, two independent groups contributed new insights into the SA-perception and signaling-cascade. Interestingly, these advances are all centered on the NPR1 protein. One study showed that NPR1 can directly bind SA and acts as an SA-receptor (Wu et al., 2012). The other group proposed that two NPR1 paralogs, NPR3, and NPR4, bind SA and control the proteasome-mediated degradation of NPR1 through their interaction with NPR1 (Fu et al., 2012). Both groups however, demonstrated the indispensable role of NPR1 in SA signaling. The focus of this perspective centers on NPR1 as the mediator of SA-perception, while comparing the SA-binding properties and molecular mechanisms of NPR1, NPR3, and NPR4. These data are compiled in **Table 1**. Furthermore, we will address some shortcomings in our current understanding of the SA-signaling pathway in the context of plant immunity.

**Table 1:** Comparison of salicylic acid (SA)-binding properties between NPR1, NPR3, and NPR4.

	NPR1	NPR3	NPR4
Method used to study SA-binding	Equilibrium dialysis (Non-equilibrium methods not working suggesting fast on/off rates)	Conventional non-equilibrium ligand binding assay (Slow off rates)	Conventional non-equilibrium ligand binding assay (Slow off rates)
Affinity	Kd = 140 ± 10 nM (High affinity)	Kd = 981 ± 409 nM (Low affinity)	Kd = 46.2 ± 2.35 nM (High affinity)
Secondary binding method	Scintillation proximity assay (Wu et al., 2012). Surface Plasmon Resonance, photoaffinity labeling, and size-exclusion chromatography (Manohar et al., 2015)	No	No
Ligand interface	Cys^521/529^	Not known	Not known
Stoichiometry	**-SA:** Oligomer **+SA: ** Dimer	Not known	**-SA: **Tetramer **+SA: ** Tetramer
Conformation change and molecular properties after SA-binding	**-SA:** N-terminal BTB domain interacts with C-terminal transactivation domain to inhibit the transcription activity of NPR1. **+SA: ** Disruption of the interaction between BTB and C-terminus converting NPR1 into a transcription co-activator.	No conformation change known. **-SA: **Does not interact with NPR1. **+SA:** Interacts with NPR1.	No conformation change known. **-SA: **Interacts with NPR1. **+SA:** Does not interact with NPR1.
Metal requirement for SA-binding	Requires copper	No	No
Crystal structure	Not determined	Not determined	Not determined

## NPR1 at the Core of the SA-Signaling Network

NPR1 is a central regulator of plant immunity, which controls both local resistance and SAR. Plants lacking a functional NPR1 protein are unable to undergo SAR or express the SAR-marker gene *PR1*, and as a result succumb to biotrophic pathogenic challenges (Cao et al., 1994; Delaney et al., 1995). Later, it was shown that NPR1 is a transcription coactivator (Rochon et al., 2006). The molecular mechanisms of NPR1 function are best understood in the case of *PR1.* Transcription of *PR1* is repressed by TGA2 transcription factor under SA concentration existing in naïve cells (Zhang et al., 2003; Rochon et al., 2006). Upon build-up of SA, NPR1 activates *PR1* transcription by forming an enhansome with TGA2 on the promoter and negating the repressor activity of TGA2 (Boyle et al., 2009). The formation of the enhanceosome is well understood. However, the exact role played by SA leading to its formation remains unclear.

Structurally, NPR1 contains an N-terminal BTB/POZ domain, an ankyrin repeat domain, a C-terminal transactivation domain and a nuclear localization sequence. The ankyrin repeats of NPR1 are responsible for its interaction with TGA2 (Zhang et al., 1999). The BTB/POZ also contacts TGA2 masking its repressor domain (Boyle et al., 2009). Besides its role in converting TGA2 from a repressor to an activator, the BTB/POZ also acts as an autoinhibitory domain. In the absence of SA, it interacts with the NPR1 C-terminal transactivation domain, and inhibits the transcription co-activator function of NPR1 (Wu et al., 2012). Two cysteines (Cys521 and Cys529), located in the C-terminus of NPR1, are crucial for the SA-induced transactivation activity of NPR1 (Rochon et al., 2006). These same Cys are required for the direct binding of SA to *Arabidopsis* NPR1 (Wu et al., 2012). Mechanistically, the binding of SA leads to the disruption of the interaction between the BTB/POZ and the C-terminus, thus releasing the C-terminal transactivation domain from autoinhibition by the BTB/POZ domain and converting NPR1 into an activated transcription co-activator.

A novel and interesting feature of NPR1, aside from being a newly discovered and important phytohormone-receptor, is the requirement of the transition metal copper for SA-binding. Mutation of Cys521 and Cys529 of the C-terminal transactivation domain not only disrupts the SA-binding capacity of NPR1, but also eliminates the recruitment of copper by NPR1 (Wu et al., 2012). This is the first plant example of a copper-binding protein acting as a transcription regulator. The fact that NPR1 is a metalloprotein explains why it took so long to identify it as an SA receptor. Many researchers, by default, include EDTA as a chelator when preparing buffers. However, recruitment of SA by NPR1 is EDTA-sensitive and its presence in buffers precludes SA from binding to NPR1 (Wu et al., 2012). Despite the fact that NPR1 is the first copper-binding transcription-regulator discovered in plant, it is not the first time that copper is found to play a critical function in hormone signal-transduction pathway. The high-affinity binding-activity of the gaseous plant hormone, ethylene, to the ethylene receptor, ETR1, also requires copper as a cofactor (Rodriguez et al., 1999). As is the case of SA in NPR1, ethylene is coordinated to copper in the ETR1 hormone-binding pocket.

## NPR3 and NPR4: The Newer Kids on the Block

NPR1 is a positive regulator of SAR. Recently, additional members of the NPR family, NPR3, and NPR4, were shown to negatively regulate SAR (Liu et al., 2005; Zhang et al., 2006; Fu et al., 2012). Analysis of conceptual gene products revealed that NPR3 and NPR4, respectively, share 34.5 and 36.0% amino acid-conservation with NPR1, specifically in the BTB/POZ and ankyrin repeat domains (Liu et al., 2005). Protein alignments indicate that all three NPR share four (4) conserved Cys in their BTB/POZ domain, and a stretch of five (5) variable basic-amino acids at the C-terminal, that may be involved in nuclear localization (Shi et al., 2013). The structural similarities among these three protein appears to extend to their functional roles including SA-perception and interaction with members of the TGA family of transcription factors (Després et al., 2000; Kinkema et al., 2000; Subramaniam et al., 2001; Fan and Dong, 2002; Mou et al., 2003; Rochon et al., 2006).

At the organ level, expression of NPR1/NPR3/NPR4 appears to occur in different locations. Promoter-driven GFP expression observed with fluorescence stereomicroscopy, demonstrated that NPR1 was detectable only in leaves, NPR4 only in mature siliques and roots, while NPR3 was expressed in relatively high quantities in the young flower (Shi et al., 2013). At the subcellular level, NPR3/NPR4-TGA2 interactions have been observed primarily in the nucleus, when studied in onion epidermal cells and *Arabidopsis* mesophyll protoplasts (Zhang et al., 2006). While nuclear localization of NPR1 has been shown definitively, differing reports have suggested that NPR1 can also be observed in the cytoplast as well (Després et al., 2000).

The pathology surrounding *npr1/npr3/npr4* mutants has displayed different phenotypes under the exact and differential conditions. Early experiments infecting *npr4-1* plants with the fungi *Erysiphe cichoracearum* (powdery mildew) and bacterium *Pseudomonas syringae* pv*. Tomato DC3000* (*Pst* DC3000) indicated that these plants were compromised in disease resistance (Liu et al., 2005). However, an independent study from Zhang et al. (2006), partially disagreed, rather observing that the *npr4-3* and *npr4-2* plants were not more susceptible to *Pst* DC3000 or *P. syringae* pv. *maculicola* ES4326 (*Psm* ES4326). When combined with the *npr3-1* mutant (*npr3-1npr4-3)* plants were found to be more resistant (Zhang et al., 2006). Corroborating the results of Zhang et al. (2006), single *npr3* or *npr4* mutants showed little difference in SAR response when compared to Col-0. Furthermore, the double mutant (*npr3npr4)* was highly resistant in basal and induced SAR states (Fu et al., 2012). At the basal level, NPR3 deficient backgrounds have compromised fitness when measured by primary root length, average growth rates, and seed production. Most recently an *npr3-3* mutant was generated and found to not differ from Col-0 plants in terms of quantity of bacterial growth when leaves were infiltrated with *Pst* DC3000, consistent with previous data. Conversely, transgenic plants overexpressing NPR3 were more susceptible to inoculation (Shi et al., 2013). Interestingly, the quantity of NPR3 transcripts was approximately threefold lower in flower petals when taken from the *npr3-3* background in comparison to the *npr3-2* mutant (Shi et al., 2013). Although, both backgrounds were created from homozygous T-DNA insertions in the third exon, the *npr3-2* plant may nonetheless be a “weak allele” in flowers, at least (Shi et al., 2013). The discrepancies observed between laboratories when testing the same pathogens reflect the complexity of the disease resistance phenotype compared to the analysis of the SAR-marker gene *PR1*. Differences may result from the use of different mutant alleles. However, the theme emerging from these data is the functional redundancy, at least in leaves, of NPR3 and NPR4 with respect to immunity. This somewhat contrasts with the proposed role of NPR4 and NPR3 functioning as independent SA-receptors under low and high SA concentrations, respectively.

## NPR1/NPR3/NPR4, SA and the Regulation of SAR: Some Shortcomings

Contemporary analysis suggests that NPR4 is a CUL3 E3-ligase substrate-adapter in naïve cells, which can interact with NPR1, allowing for the continuous ubiquitylation and turnover of NPR1 by the proteasome. During SAR, the cellular accumulation of SA allows NPR4 to bind the hormone, disrupting the NPR4–NPR1 interaction and abolishing the adaptor-substrate complex. Conversely, NPR3 responds to the abundance of SA by presumably binding to the hormone allowing NPR3 to interact with NPR1, resuming ubiquitylation of NPR1, targeting it for degradation (Fu et al., 2012). Hence, NPR3 and NPR4 would function as both substrate adaptors and SA- receptors that mediate the degradation of NPR1 in the SAR induced and naïve cells, respectively.

Despite the attractiveness of this model, it has yet to be demonstrated how NPR3 or NPR4 actually interact and bind SA. Furthermore, no structural changes in these proteins were directly observed upon binding SA. Such conformational changes are the usual hallmark of receptor-ligand interactions. In what appears to be a controversial finding, the study by Fu et al. (2012) suggested that NPR1 was unable to bind SA. Interestingly, using the same non-equilibrium method (see **Table 1**), Wu et al. (2012) came to the same conclusion, as they also found that NPR1 could not bind SA under these conditions. However, NPR1 clearly binds SA under equilibrium conditions when appropriate methodologies are used and chelating agents are omitted from experimental buffers (Wu et al., 2012; **Table 1**). Furthermore, while this manuscript was under review, Manohar et al. (2015), demonstrated, using three alternative methods, that NPR1 binds SA, bringing to five the total number of methods tested to demonstrate that NPR1 is an SA-receptor (**Table 1**). While these data clearly confirm that NPR1 binds SA and should put an end to the controversy, they also clearly show the need to confirm that NPR3 and NPR4 can indeed bind SA, especially given the fact that they do not undergo conformational changes upon binding SA. Therefore, considering that NPR1 is also an SA-receptor that binds the hormone with a relatively high affinity in the presence of copper, it is also unclear *in vivo* whether the interaction between NPR1–NPR3/NPR4 is a result of SA bound to NPR1 or to NPR3/NPR4. Since yeast-two-hybrid assays were used to study the SA-dependent interactions between NPR1 and NPR3/NPR4, it is possible that cellular copper was present at quantities sufficient to allow NPR1 to bind SA. Transient BiFC assays in naïve onion epidermal cells have also indicated an interaction between NPR1 and NPR3. However, it is unclear whether basal levels of SA were present at sufficient concentrations in the naïve onion epidermal cells to allow SA perception by NPR1 or NPR3, making it unclear whether or not the interaction requires NPR1 bound SA, NPR3 bound SA, or whether the interaction requires the presence of SA at all *in vivo* (Shi et al., 2013). However, because NPR1 has a higher affinity for SA than NPR3, as observed by the respective dissociation constants, it would follow that NPR1 would outcompete NPR3 for the interaction with SA (Fu et al., 2012; Wu et al., 2012). Given that NPR1 is the only NPR (among NPR1/NPR3/NPR4) shown to display a conformational change upon binding to its ligand, NPR1 may in fact be the decisional entity responsible for dictating whether interaction with NPR3/NPR4 occurs, regardless of the SA-status of the system.

Although NPR3 and NPR4 appear to degrade NPR1 in an SA-dependent and independent model, respectively, the biochemical and phenotypic data observed from the *npr3, npr4,* and *npr3npr4* mutant plants are not always in agreement with this hypothesis. For example, in the *in vivo* NPR1 degradation experiment (Figure 1A in Fu et al., 2012), in the *npr4* mutant, in which the NPR3-mediated NPR1-degradation is not affected, NPR1 accumulates to the highest levels after 8 h SA application. This indicates that NPR4 and not NPR3 is responsible for degrading NPR1 under SA conditions, which is not consistent with the model. Furthermore, although NPR1 accumulates to some extent in *npr3npr4* mutant before SA application, NPR1 accumulates to even greater extent in the *npr3npr4* double mutant in response to SA treatment, which indicates that the *npr3npr4* mutant is not completely insensitive to SA, suggesting that there is(are) other SA receptor(s) which mediate or trigger the accumulation of NPR1. Another indication, illustrating the presence of central receptor(s) of SA other than NPR3/NPR4, is the data showing that *Psm* ES4326 growth is significantly decreased in the *npr3npr4* double mutant plant even without SAR induction (Figure 4A in Fu et al., 2012). This does not suggest that SAR is defective as proposed by the authors, but rather that SAR is already established in the *npr3npr4* double mutant. Further inconsistencies with the model are revealed by the SAR sets of experiments. Although SA accumulation was not quantified in these experiments, treatment with the *Psm avrRpt2* strain would presumably induce SAR and thus promote SA accumulation. Therefore the model would predict that, if NPR4 is a CUL3 substrate adaptor only in the absence of SA, the *npr3npr4* mutant should not be more resistant than the single *npr3* mutant.

On the *PR1* front, the relative expression of the gene in naïve cells shows a slightly higher than wild-type induction in the *npr3* plants and about the same induction as wild-type in *npr4* plants. By contrast *PR1* induction was several folds greater in the *npr3npr4* plants when compared with wild-type or the single *npr3* or* npr4* mutants. The current NPR3/NPR4–NPR1 degradation model would predict rather that the *npr4* plants should display similar *PR1* induction as the *npr3npr4* plants and that the *npr3* plants should be no different from the wild-type. This is expected because of the lack of NPR3-targeted degradation of NPR1 in naïve cells. As proposed by Zhang et al. (2006), NPR3 and NPR4 appear to have redundant functions with respect to immunity, as opposed to the model proposed by Fu et al. (2012), where they have distinct non-overlapping functions.

## Final Thoughts

The NPR3/NPR4-mediated NPR1 degradation is reminiscent of the emerging trend of ubiquitylation in plant hormone signaling (Santner and Estelle, 2009). Auxins act by stimulating the degradation of Aux/IAA transcriptional repressor through the ubiquitin-ligase complex SCF^TIR1^ (Gray et al., 2001). Jasmonates activate downstream gene transcription by promoting degradation of the JAZ family of repressors through SCF^COL1^ E3 ubiquitin-ligase (Chini et al., 2007). The gibberellin receptor GID1 mediates ubiquitylation and degradation of DELLA repressor, thus activating gibberellin-responsive gene transcription (Griffiths et al., 2006). It seems that in many signaling pathways, plants use ubiquitin and the proteasome pathway to regulate the abundance of negative regulators of the corresponding system. However, in contrast to the aforementioned pathways, in the case of SA signaling, the proteasome targets the positive regulator NPR1. Although the biological importance and molecular mechanism of SA-regulated NPR1-degradation needs further investigation, ubiquitylation also plays a role in mediating SA signaling (Fu et al., 2012).

Cys521 and Cys529 responsible for the binding of SA to the *Arabidopsis* NPR1 are not universally conserved in NPR1 orthologs, such as those found in crops. However, metal interaction with proteins is not limited to Cys, since any amino acid harboring electronegative elements in its side chain can potentially participate in metal interaction (Wu et al., 2012; Figure S2 therein). Objectively, this leaves us with three possible scenarios: (1) NPR1 from crops could bind SA through metal-coordination, as does the *Arabidopsis* NPR1, using amino-acids other than Cys; (2) NPR1 from crops could bind SA without coordination through a metal; (3) NPR1 from crops are not receptors for SA. Further research on crop NPR1 should prove invaluable in assessing whether, in the case of NPR1, *Arabidopsis* can serve as a model system or whether it is the exception to the rule.

Since NPR1 is a transcriptional coactivator (Rochon et al., 2006), the discovery that two NPR1 family members are Cul3 substrate-adaptors (Fu et al., 2012) came as a surprise. Given that NPR3 and NPR4, just like NPR1, interact with the TGA family of transcription factors (Liu et al., 2005; Zhang et al., 2006), a role for these proteins in transcription regulation would have been anticipated. Nevertheless, as proposed (Zhang et al., 2006), a regulatory function for NPR3 and NPR4 involving transcriptional control may still be revealed in the future.

## Conflict of Interest Statement

The authors declare that the research was conducted in the absence of any commercial or financial relationships that could be construed as a potential conflict of interest.
